# Psychological readiness is related to return to sport following hip arthroscopy and can be assessed by the Hip-Return to Sport after Injury scale (Hip-RSI)

**DOI:** 10.1007/s00167-020-06157-4

**Published:** 2020-07-22

**Authors:** Tobias Wörner, Kristian Thorborg, Kate E. Webster, Anders Stålman, Frida Eek

**Affiliations:** 1 Department of Health Sciences, Lund University, Box 157, 22100 Lund, Sweden; 2 Capio Artro Clinic, FIFA Medical Centre of Excellence, Sophiahemmet Hospital, Stockholm, Sweden; 3 Department of Orthopaedic Surgery, Sports Orthopaedic Research Center-Copenhagen (SORC-C), Copenhagen University Hospital, Amager-Hvidovre, Denmark; 4 School of Allied Health, Human Services and Sport, La Trobe University, Melbourne, Australia; 5 Department of Molecular Medicine and Surgery, Stockholm Sports Trauma Research Center, Karolinska Institutet, Stockholm, Sweden

**Keywords:** Hip arthroscopy, Return to sports, Psychological readiness

## Abstract

**Purpose:**

Psychological readiness may play an important role in the return to sport (RTS) process following hip arthroscopy (HA), but there are limited tools for the measurement of this construct. The aim of this study was to modify the Swedish version of the Anterior Cruciate Ligament-Return to Sport after Injury (ACL-RSI) scale for use in HA patients and evaluate its psychometric properties.

**Methods:**

Content validity of a modified version of the Swedish ACL-RSI (Hip-RSI) was evaluated through 127 HA patient responses and relevance ratings by an expert panel (35 patients, 9 surgeons, 11 physiotherapists). Items with low relevance were omitted. Construct validity was assessed by the association of Hip-RSI scores to hip-related sporting function (HAGOS sport) and quality of life (iHOT12). Hip-RSI scores were compared between patients who had not returned, or returned to sport participation, previous sport, and sport performance.

**Results:**

Item reduction resulted in a 6-item Hip-RSI scale with adequate content validity for the target population. Construct validity of the full and the item-reduced scale was demonstrated by correlation to HAGOS sport and iHOT12 (*r* 0.631–0.752). A gradient increase in Hip-RSI scores was found for patients returning to sport participation, previous sport, and sport performance.

**Conclusion:**

The short version of the Swedish Hip-RSI is a valid tool for the assessment of psychological readiness to RTS and can be recommended to be used in HA patients. Higher psychological readiness to RTS, assessed by the Hip-RSI, is found with increasing levels of return to sports following HA.

**Level of evidence:**

III.

**Electronic supplementary material:**

The online version of this article (10.1007/s00167-020-06157-4) contains supplementary material, which is available to authorized users.

## Introduction

Athletes with femoroacetabular impingement syndrome (FAIS) often decide to undergo hip arthroscopy with the goal to return to sport (RTS) [12]. However, just half of all athletes undergoing hip arthroscopy return to their pre-injury sport and one in five returns to previous performance levels [6, 22]. Recent research suggests that physical impairments alone cannot explain these low RTS rates, or the marked impairments in self-reported function observed in these patients [20].

Psychological factors related to autonomy (e.g., motivation) and competence (e.g., confidence, low fear) have been shown to play an important role in the RTS process [2] and should be taken into consideration during assessment of readiness to RTS [1]. In patients following anterior cruciate ligament reconstruction (ACLR), psychological readiness to RTS is strongly related to return to sport and participation at pre-injury levels of performance [19]. In the RTS process following HA, psychological readiness is also rated as one of the most influential factors by physiotherapists and surgeons in Scandinavia [21] and should, hence, be assessed.

After ACLR, psychological readiness to RTS can be assessed with the ACL-Return to Sport after Injury (ACL-RSI) scale [18], which has been translated and cross-culturally adapted into Swedish language [9]. A short, less knee-joint-specific version of the ACL-RSI (6 items) was developed to make it more accessible to other orthopaedic populations [17]. A recent study from Australia reported this short form to be a valid and reliable tool for patients following HA [8]. However, no HA patients were involved in the item reduction underlying the short form of the ACL-RSI [17] and content validity for the use on these patients can, hence, not be assumed. According to the COSMIN guidelines, content validity is the most important measurement property of a patient-reported outcome measure (PROM) [14] and should be determined when modifying a PROM for the use in a different patient population.

The purpose of this study was to validate the Hip-RSI, a modified version of the Swedish ACL-RSI, for the assessment of psychological readiness to RTS in patients following hip arthroscopy. It was aimed to adapt the full 12-item scale to the target population by performing an item reduction and to describe structural validity, internal consistency reliability, as well as content and construct validity of the full and the item-reduced scale. Associations between Hip-RSI scores and return to sport participation, previous sport, and sport performance following HA in patients with FAI syndrome further assessed the validity of the scale.

## Materials and methods

The study was approved by the Ethics committee at Lund University (DNR 2016/1068, 2019/03225) and conformed to the provision of the Declaration of Helsinki. The Hip-RSI was constructed by modifying the Swedish version of the ACL-RSI [9] and by performing an item reduction based on (1) Hip-RSI scores of patients following HA and (2) relevance rating by an expert panel (consisting of patients, surgeons performing hip arthroscopy, and physiotherapists delivering rehabilitation). Psychometric properties of the full 12-item Hip-RSI as well as the item-reduced version were described, and construct validity assessed. Validation of the new scale was further made by comparing Hip-RSI scores between patients that had returned to various levels of sport participation.

### Participants

**Fig. 1:**
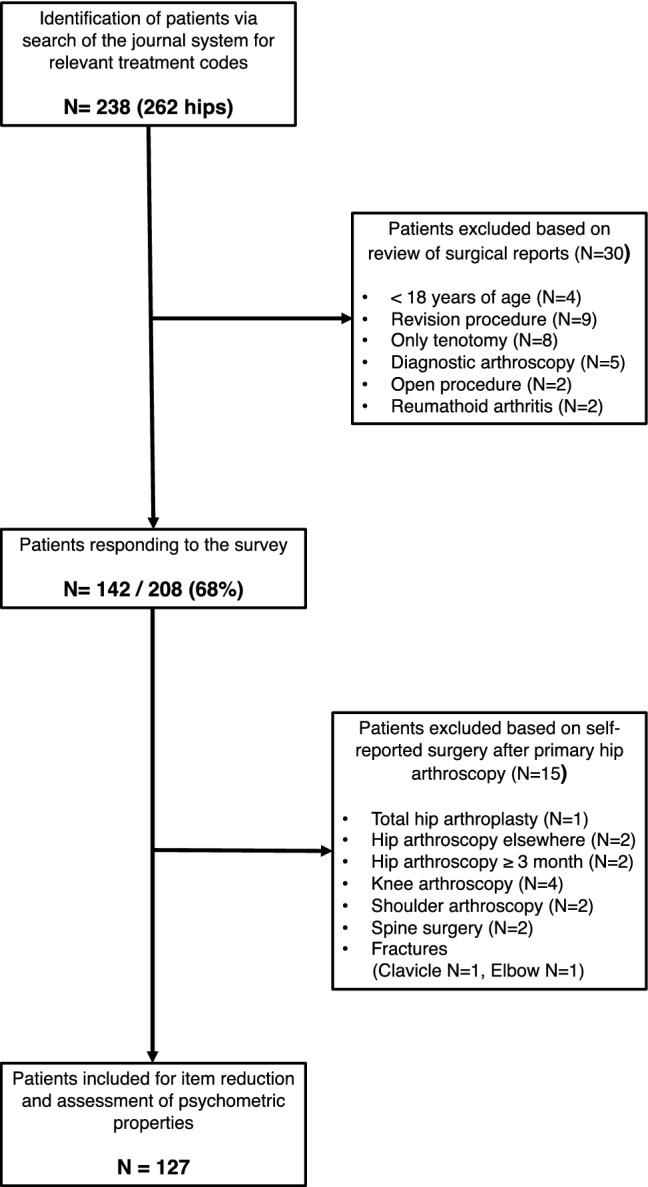
Patient flow into the study

Patients that underwent HA for the treatment of FAIS were identified via the patient register of a single surgical unit by searching for relevant diagnostic codes. Patients were included if they (a) were ≥ 18 years old; (b) had received HA for FAIS (Cam-, pincer-resection or combination) ≥ 3 months prior to data collection; (c) participated in sports/exercise prior to surgery [Hip Sports Activity Scale (HSAS) ≥ 1]; (d) did not have had any further surgery following their indexed HA. Figure 1 illustrates the patient flow into the study and Table 1 describes their characteristics. Hip-RSI scores of 127 patients (Table 1) were used for item reduction and assessment of psychometric properties.

**Table 1:** Patient characteristics (*N* = 127)

Age in years [mean (SD); range]	34.3 (10.13); 17–60
Gender [*n* (%)]	
Females	31 (24.4)
Males	96 (75.6)
HSAS pre-op (*N* = 126)	
Mean (SD)	5.5 (1.9)
Median (IQR)	5 (4–7)
Time since op (months)	
[Mean (SD); range]	19.4 (10.4); 3–39
[Median (IQR)]	18.3 (10.8–25.9)
Arthroscopic procedure (*N* = 125)	
Cam resection [*n* (%)]	109 (87.2)
Cam and pincer resection [*n* (%)]	16 (12.8)

HSAS 4 includes participation in recreational and competitive sports such as football, ice hockey, indoor sports (basketball, handball, and floorball), martial arts, and alpine sports

The expert panel included 35 different HA patients [mean time since surgery 9 months (SD 5)], 9 HA surgeons [median years of experience with HA patients 7 (IQR 2.25–12.75); median number of HA patients treated 330 (IQR 75-950)] and 11 physiotherapists [median years of experience with HA patients: 9.5 (IQR 6–10); median number of HA patients treated 50 (IQR 43-88)]. The patients included in the expert panel were identified by the same method as described above for patients responding to the Hip-RSI, recruited during a later time period. Hip arthroscopy surgeons were recruited during the Swedish hip arthroscopy meeting, held in May 2019. Physiotherapists were identified from a previous study, investigating experiences with rehabilitation following hip arthroscopy [21].

### Scale modification

The Swedish version of the ACL-RSI [9] was modified for the use on patients following hip arthroscopy by replacing the word “knee” by the word “hip” throughout the scale. The ACL-RSI is a 12-item scale, intended to measure three psychological responses to athletic injury thought to reflect the construct of psychological readiness: athlete’s emotions (5 items), confidence in performance (5 items), and risk appraisal (2 items). The scale has, however, previously shown to hold a unidimensional factorial structure and a mean score for all 12 items can be calculated [18]. Responses are given on a 0–100 visual analogue scale on which higher scores indicate higher psychological readiness.

### Data collection/procedure

In the first step, the 127 HA patients responded to an online survey, including the Hip-RSI (assessing current psychological readiness to RTS), current RTS status, as well as self-reported hip function. Patients provided their current RTS status according to consensus terminology [1] by answering whether they had (a) not returned to sport (did not participate in any sport or exercise) or returned to (b) participation (general participation in any sport or exercise), (c) sport (participation in previous sport or exercise on lower performance level than prior to hip symptoms), or (d) sport performance (participation in previous sport or exercise on same or higher performance level than prior to hip symptoms). Patients also reported their current hip function regarding quality of life and participation in sport, recreation, and physical activity by responding to two valid and reliable PROMS for hip arthroscopy patients—the International Hip Outcome Tool (iHOT12) and the sport subscale of the Copenhagen Hip and Groin Outcome Score (HAGOS) [7, 16]. Among other domains, the iHOT12 measures hip-related function in sports and recreational physical activities [5]. The HAGOS sport subscale measures a construct directly related to sport participation [16].

The expert panel received a public link to an anonymous online survey in which they were asked to rate the relevance of the individual Hip-RSI items. The expert panel was asked to rate the relevance of all 12 Hip-RSI items for the assessment of psychological readiness to RTS in hip arthroscopy patients with regard to the domain which they are supposed to measure. Rating was performed on a 4-point Likert scale (1 not relevant; 2 somewhat relevant; 3 quite relevant; 4 highly relevant). Furthermore, the expert panel was asked in an open question to indicate if they thought the scale was lacking items concerning aspects of specific relevance for HA patients.

### Analytical procedure

#### Data management

The Hip-RSI score was calculated as mean of the included items (scale 0–100, with 100 representing highest psychological readiness). The HAGOS subscale sport were computed as a score representing the percentages of the maximal score (100), with zero representing extreme amounts of hip and groin problems and 100 representing no hip and groin problems. iHOT12 scores are computed as the mean of the 12 items, on a scale from 0 to 100 with 0 representing the worst possible hip function and 100 representing the best possible hip function.

#### Scale reduction

The decision to retain or omit individual items was based on a combination of the patient responses and expert ratings. Means with standard deviations (SD) and medians with interquartile range (IQR) were computed for each item. The proportion of responses that were the minimum and maximum score (0 and 100) is reported for each item. A floor or ceiling effect is considered present if > 20% of participants score the minimum or maximum value. Within each domain, items were retained if at least two of the following criteria were fulfilled: (a) patients’ responses demonstrated central tendencies close to the center of possible range and large spread (in relation to other items in the three respective domains), and/or the item demonstrated high relevance based on (b) expert rating (mean relevance score exceeding two-thirds of maximum score, corresponding to ≥ 2.7 and/or (c) at least 67% of all experts rated them to be relevant) [10, 11, 17].

#### Psychometric properties

Psychometric properties were explored and described for the full as well as for the item-reduced scale. Structural validity was assessed by confirmative factor analysis (with varimax rotation) to determine whether the items held the same factorial structure as the original ACL-RSI. Cronbach’s alpha was computed as a measure of internal consistency reliability. Floor and ceiling effects were evaluated for the individual items. Construct validity was assessed by relating HIP-RSI scores of HA patients to hip-related quality of life (iHOT12) and sporting function (HAGOS sport). Since the data contained no extreme outliers affecting the results, the strength of correlations between Hip-RSI scores and iHOT12 as well as HAGOS sport were estimated by Pearson correlation coefficients and corresponding 95% confidence intervals (CI). We expected correlations to be larger than 0.5 between these instruments and the Hip-RSI. Since iHOT12 is measuring more than just sporting-related function, we expected correlation between Hip-RSI and the HAGOS subscale sport to be stronger.

#### Association with RTS

Differences in Hip-RSI between patients that have reached different levels of RTS was explored by analysis of variance (ANOVA), with post hoc pairwise group comparisons as well as test for linearity. Significance level was set to *P* ≤ 0.05.

## Results

### Item relevance

Half of the items were rated as relevant by between 69.1 and 90.9% of the expert panel. Patient responses for those items had a mean score close to the middle of the scale. Individual item scores as well as relevance ratings are presented in Table 2. Based on patients’ responses and expert ratings of item relevance, six items were omitted from the 12-item scale due to low face validity for the assessment of patients following hip arthroscopy. Three members of the expert panel commented that the scale is lacking items related to fear of pain during sport participation, and concerns about long-term consequences for hip health with sport participation.

**Table 2:** Patient scores and expert relevance score for individual Hip-RSI items

Scale item	Patient scores				Relevance rating	
	Mean (SD)	Median (IQR)	Floor effect (%)	Ceiling effect (%)	Mean (SD)	Rated relevant (%)
*Emotions*						
1. *Are you nervous about playing your sport?*	61.6 (30.9)	*65.5* (*33.8*–*93.9*)	3	11	2.8 (1.1)	*58.2*
2. **Do you find it frustrating to have to consider your hip with respect to your sport?**	45.9 (37.0)	41.0 (7.3–85.8)	12	10	3.3 (1.0)	80.0
3. **Do you feel relaxed about playing your sport?**	62.3 (31.8)	*67.5* (*33.8*–*93.0*)	4	14	3.0 (0.8)	70.9
4. *Are you fearful of reinjuring your hip by playing your sport?*	59.1 (32.1)	*67.0* (*31.5*–*87.5*)	4	13	3.1 (0.9)	*56.4*
5. *Are you afraid of accidentally injuring your hip by playing your sport?*	66.1 (31.8)	*77.0* (*36.0*–*95.8*)	2	17	*2.5* (*1.0*)	*45.5*
*Confidence in performance*						
6. *Are you confident that your hip will not give way by playing your sport?*	59.7 (33.9)	*61.0* (*30.0*–*93.0*)	6	12	2.7 (0.9)	*63.6*
7. **Are you confident that you could play your sport without concern for your hip?**	49.3 (34.7)	48.0 (18.5–85.5)	11	6	3.1 (0.9)	76.4
8. *Are your confident about your hip holding up under pressure?*	60.3 (31.3)	*64.0* (*34.0*–*90.0*)	4	10	2.7 (0.9)	*53.7*
9. **Are you confident that you can perform at your previous level of sport participation?**	52.7 (38.8)	52.0 (13.8–97.0)	11	20	3.4 (7.1)	90.9
10. **Are you confident about your ability to perform well at your sport?**	54.1 (35.0)	50.0 (20.0–93.5)	6	13	3.1 (0.8)	74.1
*Risk appraisal*						
11. **Do you think you are likely to reinjure your hip by participating in your sport?**	*62.3 (31.6)*	*71.0* (*38.0*–*91.0*)	3	15	2.8 (1.1)	69.1
12. *Do thoughts of having to go through surgery and rehabilitation again prevent you from playing your sport?*	*75.7 (30.1)*	*91.0* (*53.0*–*100*)	2	35	*2.5* (*1.2*)	*54.5*

Range of answer scores was 0–100 for all items. Final items with adequate relevance are marked in **BOLD**. Items to be omitted are marked in *ITALICS.* Respective patient scores as well as relevance rating underlying the decision to omit an item marked in *ITALICS* and underlined

### Psychometric properties

**Fig. 2:**
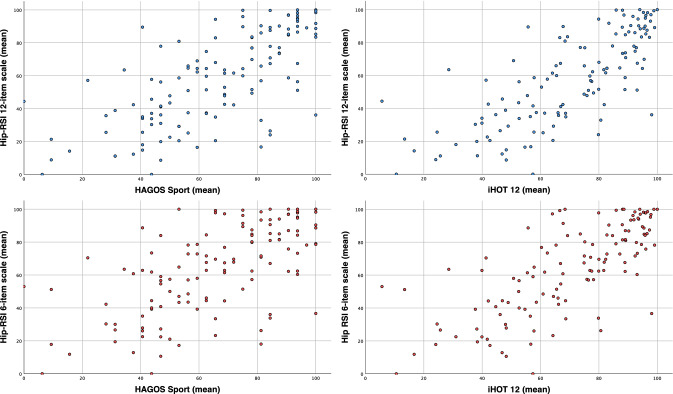
Correlations between 12-item Hip-RSI (top row/blue) as well as 6-item Hip-RSI (bottom row/red) and HAGOS sport (left) as well as i-Hot 12 (right)

Results of principal component factor analysis showed a single underlying factor accounting for 67.7% of the total variance (eigenvalue 8.1) for the full 12-item scale and 67.7% of the total variance (eigenvalue 4.1) for the 6-item scale. Cronbach’s alpha for the full 12-item scale was 0.96 and 0.90 for the 6-item scale. No floor or ceiling effects were observed for either the full or item-reduced scale (full scale: minimum score 1.4%, maximum score 1.4%; item-reduced scale min score 1.4%, max score 4.9%). In accordance with a priori hypotheses, correlations between the full as well as the short form of the Hip-RSI and HAGOS sport and iHOT12 were larger than 0.5 (Table 3 and Fig. 2).

**Table 3:** Correlations [Pearson (95% CI)] between the Hip-RSI hip function

	12-Item scale	6-Item scale
HAGOS sport	0.69 (0.66–0.96)	0.63 (0.56–0.87)
iHOT-12	0.75 (0.78–1.07)	0.73 (0.72–1.01)

*HAGOS* Hip and Groin Outcome Score, *iHOT-12* International Hip Outcome Tool

### Association with RTS

**Fig. 3:**
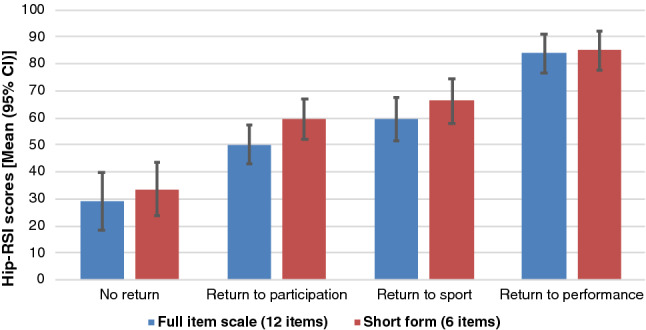
Patients’ (*N* = 127) Hip-RSI scores according to RTS-level

Higher Hip-RSI scores were found with increasing level of RTS for both the 12-item scale as well as the 6-item scale (Fig. 3), with a statistically significant linear trend (*P* < 0.001). Hip-RSI scores of RTS groups differed significantly from each other except for patients who reported return to a different sport and patients who reported return to the same sport at a lower performance levels (Table 4).

**Table 4:** Differences in Hip-RSI groups between RTS groups

Level of return to sports Pairwise comparison	Hip-RSI scores			
	12-Item scale		6-Item scale	
	Mean diff. (95% CI)	*P*	Mean diff. (95% CI)	*P*
Same sport: same perf.				
No sport/exercise	54.8 (35.3 to 74.2)	< 0.001	51.4 (31.8 to 71.1)	< 0.001
Different sport/exercise	34.0 (19.8 to 48.1)	< 0.001	25.5 (11.2 to 39.8)	< 0.001
Same sport: lower perf.	24.5 (9.5 to 39.6)	< 0.001	18.6 (3.4 to 33.8)	0.001
Same sport: lower perf.				
No sport/exercise	30.2 (11.6 to 48.8)	< 0.001	32.9 (14.1 to 51.6)	< 0.001
Different sport/exercise	9.4 (− 3.5 to 22.4)	n.s	6.9 (− 6.2 to 20.0)	n.s
Different sport/exercise				
No sport/exercise	20.8 (2.9 to 38.7)	0.016	25.9 (7.9 to 44.0)	0.002

*Hip-RSI* Hip return to sport following injury, *per.* performance

## Discussion

In this study, psychometric properties of the Swedish ACL-RSI, modified for the use in patients undergoing hip arthroscopy, were assessed and an item-reduction based on patient responses and expert rating was performed. The item-reduced, 6-item version of the Hip-RSI was found to be an internally consistent, unidimensional, and valid tool for the assessment of psychological readiness to RTS after arthroscopic treatment of FAI syndrome in physically active patients. Psychological readiness to RTS, assessed by the Hip-RSI, was gradually greater as patients had returned to participation, previous sports, and performance.

This is the first study investigating content validity of a hip-modified ACL-RSI version for the assessment of psychological readiness to RTS in patients following HA. Arthroscopic treatment of ACL ruptures aims to restore knee stability, but athletes frequently decide not to RTS, because they experience recurrent knee instability and fear reinjury [15]. Arthroscopic treatment of FAI syndrome, on the other hand, aims to reshape hip morphology to reduce mechanical impingement [4], and the main reason not to RTS appears to be lingering pain [6]. These fundamental differences are reflected in the item-reduction process. The short form of the ACL-RSI [17] has previously been tested on HA patients [8]. However, our scale modification and item-reduction process was based on responses and opinions from the target population of HA patients. The resulting short form of the Hip-RSI, hence, differs from the short form of the ACL-RSI. In direct comparison of the two versions, the Hip-RSI presented in this study does focus less on joint instability and fear of reinjury while putting more emphasize on confidence in performance. The HA patient population-based item-reduction process resulted in a 6-item Hip-RSI scale with adequate content validity for the use in HA patients. Performance and injury-related fears, anxiety, and confidence are reported to be associated with RTS [3] and these aspects are covered by the items included in the 6-item Hip-RSI version.

The Hip-RSI was found to be correlated to self-reported hip and groin function in the direction and magnitude specified in the a priori hypothesis regarding construct validity. While HAGOS sport measures specific hip-related sporting function, iHOT12 assesses hip-related quality of life [5, 16]. Contrary to our expectations, we did not find stronger correlations between the Hip-RSI and HAGOS sport compared to iHOT12, which suggests that psychological readiness to RTS is affected by more than just joint-specific physical recovery. In ACL patients, thigh muscle strength and jump testing has been found to have little-to-no association to psychological readiness to RTS [13], further pointing towards the need to assess and treat both physical and psychological recovery following surgery. In this study, a gradient increase in Hip-RSI scores was found with increased level of RTS, further strengthening the construct validity of the scale. The Hip-RSI showed discriminant validity by yielding different scores for patients that made no return, returned to previous sports, and returned to sport performance. Hip-RSI scores of patients changing sports and returning to the previous sport on lower performance levels did not different significantly, further highlighting the importance of items assessing performance-related fears, anxiety, and confidence, which have shown to be associated with RTS [3] and rated to be highly relevant by our expert group. Hence, results of this study further highlight the relationship between psychological readiness to RTS and actual level of return to sports, but, most importantly, present a valid tool for the assessment of psychological readiness in patients following HA for FAI syndrome.

There are a number of methodological considerations to make when interpreting the current study. The current study investigated psychometric properties of a hip-modified version of the Swedish ACL-RSI version [9] and it cannot be assumed that results transfer directly to the English version. The sample of this study consisted of a homogeneous group of patients in terms of surgical indication and arthroscopic treatment. All participants underwent HA for FAI syndrome and results of this study can, hence, be generalized to this group of patients. Patients answered the Hip-RSI at various follow-up times, ranging from 3 to 39 months following surgery. Psychological readiness may differ for patients at different follow-up times. The survey is intended to be applicable at different time points during the rehabilitation period. The potential spread in Hip-RSI results was, hence, warranted by our primary aim to investigate its psychometric properties not only at a specific time point but during the longer period between surgery and RTP. Future prospective studies should investigate the trajectory of psychological readiness to RTS after HA for FAI syndrome, preferably alongside collecting data about the recovery of physical function as well as return to sport. The ACL-RSI is intended to measure psychological readiness to return to sports in ACL patients. The stringent item-reduction process applied in this study can be expected to have excluded items with low relevance for HA patients. Conversely, there might be aspects of psychological readiness important to HA patients that are not included in the original ACL-RSI and, hence, neither in the Hip-RSI. Future studies should consider adding items assessing aspects where highlighted by experts in this study, such as fear of pain during sport participation and concerns about future hip health upon RTS. According to the COSMIN guidelines, content validity, which is assessed in this study, is the most important measurement property of a patient-reported outcome [14]. Following the COSMIN guidelines, content validity of the Hip-RSI was assessed by involving patients and other relevant medical professionals that rated relevance of the different items. Due to the cross-sectional design of this study, additional psychometric properties such as test–retest reliability, responsiveness, and measurement error of the Hip-RSI were not described in this study. The study by Jones et al. [8] reported that the short ACL-RSI showed excellent test–retest reliability and responsiveness to change in HA patients. It can be expected that the short form of the Hip-RSI, containing only items with relevance for HA patients, will demonstrate similar or even better test–retest reliability and responsiveness to change. However, these psychometric properties of the short 6-item Hip-RSI have to be evaluated prospectively in future studies.

## Conclusion

The hip-modified and item-reduced version of the Swedish ACL-RSI (Hip-RSI) demonstrated adequate validity for the assessment of psychological readiness for return to sport in HA patients. The Hip-RSI was able to discriminate between patients that returned to their previous sports and sport performance, highlighting the potential impact of psychological aspects in the RTS process and, hence, the need to assess and address psychological readiness to RTS in this group of patients.

## Electronic supplementary material

Below is the link to the electronic supplementary material.

Supplementary material 1 (PDF 165 kb)
