# Supplementary material for: Psychological readiness is related to return to sport following hip arthroscopy and can be assessed by the Hip-Return to Sport after Injury scale (Hip-RSI)
Source: Knee Surg Sports Traumatol Arthrosc. 2020 Jul 22;29(5):1353–61. doi: 10.1007/s00167-020-06157-4 (PMC8038984; doi:10.1007/s00167-020-06157-4)
Supplement: Supplementary file 1 — Supplementary material 1 (PDF 165 kb) [file 167_2020_6157_MOESM1_ESM.pdf]

Hip-RSI (short version) – English version

Instructions: Please answer the following questions referring to your main sport prior to injury. For each question, please mark a vertical line through the horizontal line to indicate how you feel right now relative to the two extremes.

1. Are you confident that you can perform at your previous at your previous level of sport participation?

Not at all confident ______________________________ *Fully confident*

2. Do you think you are likely to reinjure your hip by participating in your sport?

Extremely likely ______________________________ *Not likely at all*

3. Are you confident that you could play your sport without concern for your hip?

Not at all confident ______________________________ *Fully confident*

4. Do you find it frustrating to have to consider your hip with respect to your sport?

Extremeley frustrating ______________________________ *Not at all frustrating*

5. Are you confident about your ability to perform well at your sport?

Not at all confident ______________________________ *Fully confident*

6. Do you feel relaxed about playing your sport?

Not at all relaxed ______________________________ *Fully relaxed*

NB! Validation of the short Hip-RSI has only been performed on the Swedish version

Hip-RSI (short version) – Swedish version

Instruktioner: Vänligen svara på följande frågor med tanke på den huvudsakliga idrottsaktivitet du utövade innan skadan. Besvara varje fråga genom att markera med ett kryss på linjen, som beskriver hur du upplever situationen just nu i relation till de två ytterligheterna.

1. Är du säker på att du kan utöva din idrottsaktivitet på samma nivå som tidigare?

Inte alls säker ________________________________ *Helt säker*

2. Tror du det är sannolikt att du skadar din höft igen genom att delta i din idrottsaktivitet?

Extremt sannolikt ________________________________ *Inte alls sannolikt*

3. Är du säker på att du kan utöva din idrottsaktivitet utan bekymra dig för din höft?

Inte alls säker ________________________________ *Helt säker*

4. Upplever du att det är frustrerande att behöva ta hänsyn till din höft med avseende på din idrottsaktivitet?

Extremt frustrerande ________________________________ *Inte alls frustrerande*

5. Är du säker på din förmåga att kunna prestera bra i din idrottsaktivitet?

Inte alls säker ________________________________ *Helt säker*

6. Känner du dig avspänd inför att utöva din idrottsaktivitet?

Inte alls avspänd ________________________________ *Helt avspänd*
